# Volumetric MRI-Based Biomarkers in Huntington's Disease: An Evidentiary Review

**DOI:** 10.3389/fneur.2021.712555

**Published:** 2021-09-21

**Authors:** Kirsi M. Kinnunen, Adam J. Schwarz, Emily C. Turner, Dorian Pustina, Emily C. Gantman, Mark F. Gordon, Richard Joules, Ariana P. Mullin, Rachael I. Scahill, Nellie Georgiou-Karistianis, The Huntington's Disease Regulatory Science Consortium (HD-RSC), Varun Aggarwal

**Affiliations:** ^1^IXICO, London, United Kingdom; ^2^Takeda Pharmaceuticals, Ltd., Cambridge, MA, United States; ^3^Critical Path Institute, Tucson, AZ, United States; ^4^CHDI Management/CHDI Foundation, Princeton, NJ, United States; ^5^Teva Pharmaceuticals, West Chester, PA, United States; ^6^Wave Life Sciences, Ltd., Cambridge, MA, United States; ^7^Huntington's Disease Research Centre, UCL Institute of Neurology, London, United Kingdom; ^8^School of Psychological Sciences and Turner Institute for Brain and Mental Health, Monash University, Melbourne, VIC, Australia

**Keywords:** Huntington's disease, neurodegenerative, biomarkers, neuroimaging, volumetric MRI, C-Path

## Abstract

Huntington's disease (HD) is an autosomal-dominant inherited neurodegenerative disorder that is caused by expansion of a CAG-repeat tract in the huntingtin gene and characterized by motor impairment, cognitive decline, and neuropsychiatric disturbances. Neuropathological studies show that disease progression follows a characteristic pattern of brain atrophy, beginning in the basal ganglia structures. The HD Regulatory Science Consortium (HD-RSC) brings together diverse stakeholders in the HD community—biopharmaceutical industry, academia, nonprofit, and patient advocacy organizations—to define and address regulatory needs to accelerate HD therapeutic development. Here, the Biomarker Working Group of the HD-RSC summarizes the cross-sectional evidence indicating that regional brain volumes, as measured by volumetric magnetic resonance imaging, are reduced in HD and are correlated with disease characteristics. We also evaluate the relationship between imaging measures and clinical change, their longitudinal change characteristics, and within-individual longitudinal associations of imaging with disease progression. This analysis will be valuable in assessing pharmacodynamics in clinical trials and supporting clinical outcome assessments to evaluate treatment effects on neurodegeneration.

## Introduction

Huntington's disease (HD) is an autosomal-dominant inherited neurodegenerative disorder characterized by motor impairment, cognitive decline, and neuropsychiatric disturbances. The causative mutation is an expansion of a CAG-triplet repeat in the huntingtin (*HTT*) gene above a threshold that leads to cumulative toxicity driven by mutant huntingtin (mHTT) protein (1). HD is a continuous biological process and individuals experience variable rates of disease progression and constellations of symptoms.

Clinically, HD is diagnosed when a characteristic motor syndrome is recognized in the context of a known HD family history. Confirmatory genetic testing is typically obtained, particularly when the family history is equivocal. “Clinical motor diagnosis” in research usually equates to a clinician's rating of diagnostic confidence level (DCL) of “4” (on the 0–4 scale) on the Unified Huntington's Disease Rating Scale (UHDRS).

Categorical descriptors of disease stages are widely used in research, and most commonly to date the term “premanifest” has been applied to individuals with a CAG expansion who have not yet reached the criteria for HD clinical motor diagnosis and “manifest” to those with a clinical motor diagnosis. This terminology has been substituted throughout this review with more precise descriptions, but for consistency with the published literature we have kept these terms in some figures. Prior to the appearance of motor signs there are many detectable neurodegenerative, clinical, and functional changes that have been described in multiple studies (2–5). Neuropathological studies indicate that HD progression is accompanied by a characteristic pattern of brain atrophy, with the earliest and most dramatic changes evident in the basal ganglia, specifically the caudate and putamen^1^ (i.e., dorsal striatum) (6, 7). CAG age product (CAP) score provides an index of the length and severity of an individual's exposure to the effects of the *mHTT* gene and has proven to be a good predictor of pathology in the brains of HD patients at autopsy (8, 9).

The Huntington's Disease Regulatory Science Consortium (HD-RSC) is an initiative led by the Critical Path Institute and CHDI Foundation that currently has 37 member organizations from industry, academia, nonprofit organizations, and patient advocacy groups, as well as iterative participation from global regulatory agencies. The HD-RSC's overall aim is to create and advance drug development tools for regulatory endorsement that will accelerate the development of novel therapeutics for HD. Under the aegis of the HD-RSC, the imaging sub-team of the Biomarker Working Group was tasked with reviewing the available evidence linking regional brain volumetric magnetic resonance imaging (vMRI) measurements (the most relevant being those from the caudate and putamen) to the biological and clinical characteristics of HD, with a view toward assessing the utility of vMRI in HD clinical research (10). This sub-team is also preparing a manuscript discussing recommendations for the optimized use of vMRI in HD clinical research.

Here, we summarize cross-sectional associations between vMRI measures and clinical-genetic disease characteristics to underscore the face validity of these imaging measures. We also evaluate the relationship of vMRI measures with subsequent clinical change, their longitudinal change characteristics, and within-individual longitudinal associations of vMRI with disease progression. Ultimately, this longitudinal imaging data from interventional trials can help assess pharmacodynamics as well as support clinical outcomes to assess treatment effects on neurodegeneration.

## Regional Brain Volumes are Reduced in Huntington's Disease

**Figure 1:**
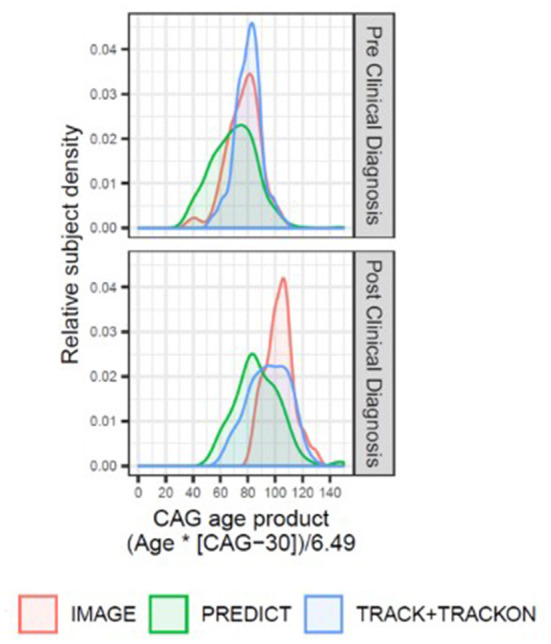
Overview of the disease progression heterogeneity for imaging studies with publicly available datasets (IMAGE-HD, PREDICT-HD, and TRACK-HD/Track-On HD) displaying the relative proportion of participants within each study (*y*-axis) by participant CAP score (*x*-axis). A single CAP score was computed for each participant using the age at enrollment in that study. The density plots are colored by study and separated by HD clinical diagnosis status (upper panel: individuals with expanded CAG repeats before clinical motor diagnosis; lower panel: individuals with HD clinical motor diagnosis). The TRACK-HD and Track-On HD had overlapping populations, and study sites, and were combined.

Notably, striatal volume loss in HD correlates with both neuronal cell loss and CAG-repeat length, as well as age at clinical motor diagnosis (6, 9, 11–14). This signature volume loss can be detected and tracked longitudinally *in vivo* using vMRI methods, as reported for several large observational studies such as PREDICT-HD (15–17), TRACK-HD (2–4, 18) and IMAGE-HD (19, 20) (Figure 1; see also Supplementary Material), as well as smaller investigations (21–23). Significantly reduced striatal volume is detectable more than 20 years prior to clinical motor diagnosis, whereas losses in other brain structures are more apparent in later disease stages (8, 24–27).

**Figure 2:**
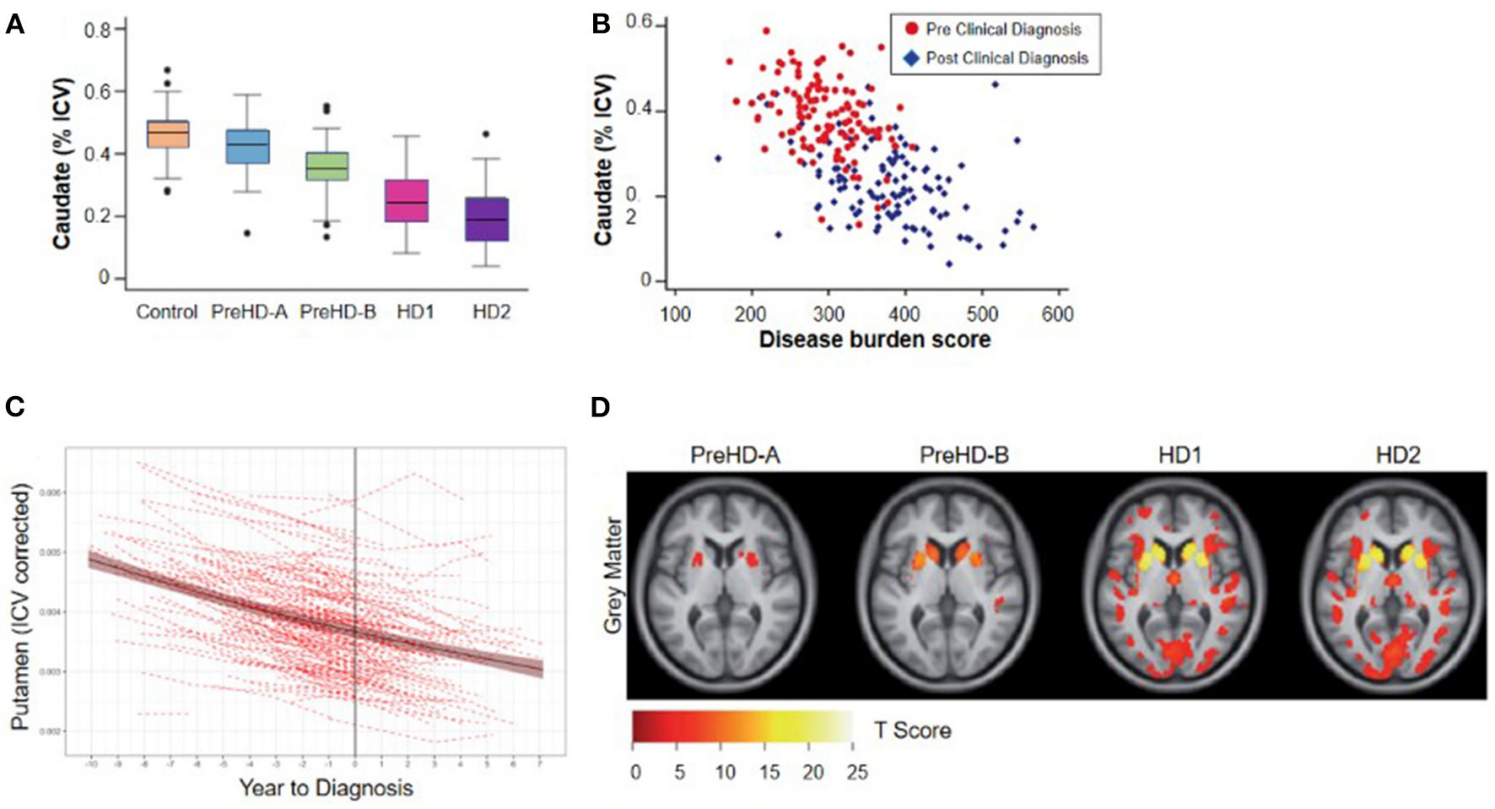
Cross-sectional relationship between regional brain volume loss from vMRI and disease progression. **(A)** Regional volume analysis shows a monotonic decrease in caudate volume as a function of TRACK-HD cohort. The group of individuals without clinical motor diagnosis at baseline were divided at the median into PreHD-A (further from predicted diagnosis) and PreHD-B (nearer to predicted diagnosis). HD1: participants with a clinical motor diagnosis and TFC 11-13; HD2: participants with a clinical motor diagnosis and TFC 7-10. **(B)** Regional volume analysis shows an inverse correlation with disease burden score [age^*^(CAG length-35.5)] over all groups of participants; similar results were found for putamen and whole striatum (4). **(C)** Trajectory of putamen volume for *N* = 225 participants who received a clinical motor diagnosis during PREDICT-HD. Shown are the individual dashed empirical curves and the solid fitted spline curve with the 95% confidence interval. The vertical line indicates the year of clinical motor diagnosis (set to year = 0). **(D)** Whole-brain VBM analysis of baseline data from the TRACK-HD shows that the strongest differences from controls are concentrated in the striatum including the caudate. The figure displays statistical parametric maps of gray matter differences in each group compared with controls with the data adjusted for age, sex, study site and intracranial volume. Results are corrected for multiple comparisons using familywise error at the *p* < 0.05 level (4).

Imaging studies in the 1990s demonstrated that reduced striatal volumes were detectable *in vivo* in the early stages of clinically-diagnosed HD (21, 23), and such volume loss has since been identified in individuals up to 24 years before clinical motor diagnosis (4, 22). Overall, the relationship between brain volume and age at clinical diagnosis appears monotonic, with smaller striatal volumes associated with decreasing time to estimated diagnosis and with increasing CAP score (9, 28) (Figures 2A,B). At the time of clinical motor diagnosis, striatal volumes are markedly reduced compared to age-matched normal volumes [e.g., caudate: 52–70% loss; putamen: 43–67% loss; nucleus accumbens: 59–60% loss (29, 30)].

**Figure 3:**
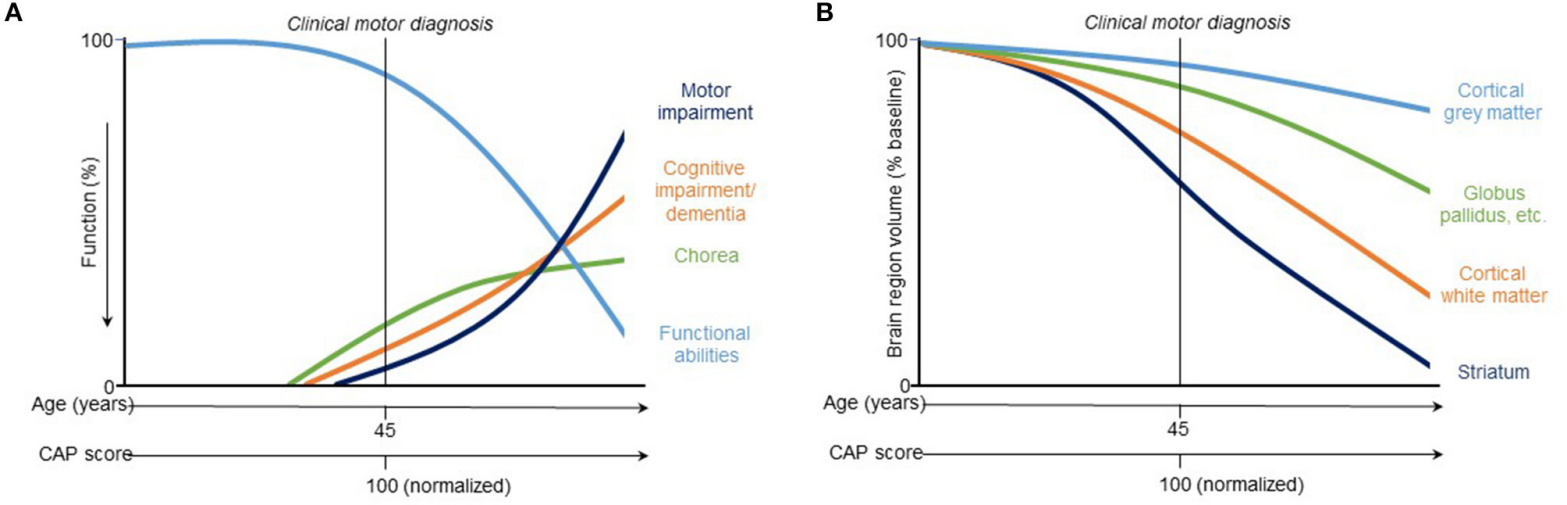
Hypothetical model of trajectories of **(A)** clinical and **(B)** vMRI measures of HD progression [modified from Ross et al. (8)]. This qualitative representation is based on observations across two observational study cohorts (PREDICT-HD and TRACK-HD) and provides a conceptual illustration for quantitative trajectory models but has not yet been experimentally confirmed.

While regional brain volume losses in the caudate and putamen can be detected many years prior to the onset of motor symptoms, loss in cortical regions becomes more widespread after clinical motor diagnosis (17, 22, 30–36) (Figures 2C,D). Nevertheless, thinning of the cortical gray matter can be detected prior to the onset of motor symptoms, even after correction for putamen and caudate volume loss (4, 37). This cortical gray matter loss is apparent in occipital, motor, dorsomedial prefrontal and parietal cortices, and becomes more pronounced with more advanced disease (4, 38). Moreover, cerebral white matter volume is reduced early in the disease [CAP score (9)> 250; TMS < 5] and continues to decrease with disease progression (4). Collectively, these findings have enabled a qualitative understanding of the regional patterns and trajectories of brain atrophy, and their relationship to clinical symptoms (Figure 3).

## Relationships Between Brain Volume Measurements and Clinical Outcome Measures

Numerous studies have demonstrated significant cross-sectional associations in HD between brain volume loss and clinical outcome measures of both motor and cognitive function. For example, in a study of individuals with expanded CAG repeats before clinical motor diagnosis, regional brain volumes correlated with both subtle motor impairments (speeded tapping and UHDRS-derived scales) and cognitive impairments (39), despite the reduced sensitivity of available clinical instruments in this population. Overall, caudate, putamen, and globus pallidus volumes had the strongest correlations with different clinical outcome measures (Table 1).

**Table 1:** Cross-sectional partial correlations between regional brain volumes and clinical outcome measures in individuals prior to clinical motor diagnosis, after removing the effect of age, sex, and years of education, obtained from an analysis of the PREDICT-HD study (39).

	**Motor (** ***N*** **=** **504)**						**Cognitive (** ***N*** **=** **486)**					**Functional (** ***N*** **=** **516)**	
	**Speeded tapping**	**UHDRS total motor**	**UHDRS oculomotor**	**UHDRS bradykinesia**	**UHDRS chorea**	**UHDRS dystonia**	**Symbol-digit modalities**	**Hopkins verbal learning**	**Emotion recognition**	**Self-paced timing**	**Letter-number sequencing**	**Total functional capacity**	**Functional assessment scale**
Putamen	−0.42	−0.27	−0.18	−0.22	−0.22		0.33	0.23	0.31	0.32	0.15		
Caudate	−0.42	−0.29	−0.2	−0.25	−0.21		0.31	0.33	0.33	0.32	0.2		
Globus pallidus	−0.36	−0.3	−0.2	−0.24	−0.27		0.35	0.29	0.33	0.34	0.18		
Thalamus	−0.14						0.12						
Nucleus accumbens	−0.2	−0.14	−0.13				0.13		0.15				
Hippocampus													
Frontal white	−0.15	−0.15	−0.12	−0.16									
Parietal white	−0.21	−0.12		−0.17			0.23		0.16	0.18	0.13		
Occipital white				−0.13			0.19		0.13				
Temporal white		−0.13		−0.17			0.12						
Frontal gray													
Parietal gray													
Occipital gray													
Frontal gray													

*Only correlations that remained statistically significant following false discovery rate multiplicity correction are shown. The directionality of the associations was such that smaller tissue volumes were associated with greater impairment (higher scores on motor tasks, lower scores on cognitive tasks). Significant positive correlations are highlighted in a red color scale, significant negative correlations in a blue color scale, and darker shades are associated with stronger correlation coefficients*.

In clinically-diagnosed HD participants (with Total Functional Capacity (TFC) ≥ 11), volumetric (manual segmentation-based) and whole-brain voxel-based morphometry (VBM) analyses revealed correlations (*p* ≤ 0.05) between performance across motor (Purdue pegboard^®^, UHDRS-TMS, Hand-arm test), cognitive (Symbol Digit Modalities Test), and everyday functioning (TFC) domains, and both caudate volume and VBM-measured tissue concentration (32). For the putamen and the globus pallidus, both volume and tissue concentration only correlated (*p* ≤ 0.05) with performance on two of the motor assessments (Purdue pegboard^®^, UHDRS-TMS), and thalamus tissue concentration, but not volume, showed correlations (*p* ≤ 0.05) with performance on the Purdue pegboard^®^, UHDRS-TMS, TFC, and Symbol Digit Modalities Test (32). Another VBM study (40) found inverse correlations (partial, corrected for age and CAG-repeat length) for the UHDRS-TMS with tissue concentration in regions localized in the caudate nuclei (left *r* = −0.68, *p* = 0.00001; right *r* = −0.65, *p* = 0.0001) and adjacent internal capsule (left and right *r* = −0.72, *p* = 0.00001), as well as in the occipital lobes (left *r* = −0.72, *p* = 0.00001; right *r* = −0.65, *p* = 0.0001), cerebellum (left *r* = −0.67, *p* = 0.0001; right *r* = −0.60, *p* = 0.0001) and lower brainstem (*r* = −0.72, *p* = 0.00001). Correlations of regional brain volumetric losses with more specific measures of motor function have also been reported, including finger tapping (41), oculomotor function (42), and variability in tongue force (42).

There is considerable evidence that cognitive function is also affected early in the course of HD progression, with reduced volumetric changes correlating with declines in cognitive performance (18, 43, 44). For example, although both putamen and caudate volumes in individuals before clinical motor diagnosis were found correlated with cognitive measures that have a motor output component, caudate volumes correlated more strongly than putamen volumes with measures of verbal learning and letter-number sequencing, tasks that require executive control (39) (see Table 1). The role for striatal volume loss, particularly in the caudate, in executive functional impairment is corroborated by a VBM analysis that found correlations between the caudate [showing the most significant differences between clinically-diagnosed HD (with average UHDRS-TMS = 15.7) and age-matched control participants] and executive dysfunction on three different measures (Tower of Hanoi: *r* = 0.647, *p* < 0.001; STROOP: *r* = 0.503, *p* < 0.01; modified Wisconsin Card Sorting Test: *r* = 0.452, *p* < 0.05) (45). In the involvement of the globus pallidus in HD motor, cognitive and behavioral symptoms has so far received less attention in the vMRI literature than the caudate and the putamen, but has recently been reviewed elsewhere in the context of neuropathology (46).

As mHTT aggregates predominantly accumulate in the cortex, which is highly interconnected with the striatum (47, 48), cortical degeneration is also likely to contribute to the presentation of HD signs and symptoms. Frontal lobe volume loss generally correlates with poorer performance on tests of executive function, but can also predict other types of cognitive dysfunction (44, 49). Total frontal lobe volume, including frontal lobe gray and white matter volumes, was found to be significantly reduced in moderately-affected clinically-diagnosed individuals (mean composite UHDRS score of 123.5) but not in those who were mildly affected (mean composite UHDRS score of 66.6), suggesting that frontal lobe degeneration occurs later in the disease process (50). Table 2 highlights some of the cross-sectional correlations from the literature that have been observed between distinct areas of neurodegeneration and measures of cognitive and motor function.

**Table 2:** Areas of neurodegeneration and their correlations with functional domains/measures.

**Study**	**Participants (Relative to clinical diagnosis)**	**Area of neurodegeneration**	**Correlated functional domain/measure**
Douaud et al. (32)	Clinically diagnosed HD	Caudate (indicated by both manual segmentations and VBM)	Motor (Purdue pegboard^®^, UHDRS-TMS, Hand-arm test), cognitive (Symbol Digit Modalities Test), everyday functioning (TFC)
Douaud et al. (32)	Clinically diagnosed HD	Putamen, globus pallidus (indicated by both manual segmentations and VBM)	Motor (Purdue pegboard^®^, UHDRS-TMS)
Jech et al. (40)	Clinically diagnosed HD	Caudate, internal capsule, occipital lobes, cerebellum, brainstem (VBM)	Motor (UHDRS-TMS)
Bechtel et al. (41)	Before HD clinical motor diagnosis, Clinically diagnosed HD	Caudate, putamen, superior temporal, precentral, internal/external capsule, subgyral frontal white matter (VBM); Occipital, parietal, and primary motor cortical thickness	Finger tapping (variability of tap durations and inter-onset intervals)
Scahill et al. (42)	Before HD clinical motor diagnosis, Clinically diagnosed HD	Striatal, occipital (VBM)	Quantitative motor (tongue force), oculomotor (anti-saccade error rate)
Starkstein et al. (44)	Clinically diagnosed HD	Caudate, frontal, and left sylvian cistern atrophy	Memory/speed-of-processing (based on factor analysis of neuropsychological test scores)
Peinemann et al. (45)	Clinically diagnosed HD	Caudate (VBM)	Executive function (Tower of Hanoi, STROOP, modified Wisconsin Card Sorting Test)
Bäckman et al. (49)	Clinically diagnosed HD	Frontal region volume	Memory, executive function (Trail Making Test—part B, Rey-Osterrieth Complex Figure—memory, Word Recall, Perseverative Errors)
Aylward et al. (50)	Clinically diagnosed HD	Frontal lobe volume, frontal lobe gray and white matter volume	General cognitive functioning (Mini Mental State Examination, general verbal and spatial ability)–the correlations did not survive correction for overall brain volume loss

While the data summarized in this section support a role for striatal volume loss in executive and motor dysfunction, it is unlikely that structural degradation of these regions is the sole contributor given the shared variance and distributed nature of brain function. From a functional perspective, the caudate is implicated in supporting executive function as well as goal-directed actions and reward prediction. Regarding the well-known role of the basal ganglia in sensorimotor function, the caudate and putamen both play a role in the regulation of movement (51–53). However, the caudate may also contribute to volitional movements through the excitation of schemas and goals needed to successfully execute an action, while the putamen is particularly implicated in stimulus-action responses or the habit-based preparation and execution of actions (54, 55). That the two structures primarily subserve different aspects of sensorimotor function could explain why vMRI measurements from each may most strongly correlate with different cognitive and motor outcome measures, or may correlate with some, but not other, measures within these domains. The differential correlations observed between brain structures—such as the caudate or putamen—and clinical/functional measures across different HD stages may reflect that while both change with disease progression, they do so differentially. While these cross-sectional associations build confidence that vMRI markers have a neuroanatomically-based relationship to HD progression and thereby contribute to their face validity, understanding within-individual longitudinal relationships is particularly important (see section MRI Brain Volume Measurements Have Robust Longitudinal Change Characteristics).

## Brain Volume Measurements Predict Subsequent Clinical Progression

The value of HD prognostic biomarkers is in their ability to accurately predict, at the individual level, either the occurrence of clinical motor diagnosis, the rate of disease progression, or other relevant milestones in the disease course. There is evidence that striatal volumes correlate with CAG-repeat length and that vMRI metrics can provide additional prognostic power (56). For example, TRACK-HD illustrated that baseline caudate, putamen, and gray matter volume provided additional predictive value (beyond age and CAG-repeat length) for clinical progression (3), defined in this study as an increased TMS score of five points or more, any TFC decline, or a new DCL score of 4; baseline measures also predicted subsequent decline in TFC score in individuals with clinical diagnosis. TRACK-HD also investigated baseline voxel-wise gray matter to identify individuals (before or after clinical diagnosis) at higher risk of 4-year motor deterioration and revealed a pattern, primarily localized in the inferior caudate, that increased the cross-validated prognostic accuracy by 13% (75% to 88%) compared to using baseline clinical and genetic metrics alone (57). Furthermore, an early analysis of PREDICT-HD showed that baseline caudate volume predicted clinical motor diagnosis within 2 years with 100% accuracy (58). Subsequently, a 12-year analysis assessed the ability of 40 mixed predictors, including imaging, motor, and cognitive measures, to predict time to CAG-adjusted age of clinical motor diagnosis. In this joint model of longitudinal and survival data, the strongest predictors of clinical motor diagnosis (16) (beyond CAG-repeat length and age) in each domain were TMS (motor), putamen volume (imaging), and Stroop word test (cognitive).

Further evidence to support caudate volume as a robust predictor of HD clinical motor diagnosis comes from a recent study (59) that used a multivariate machine learning approach in combination with permutation modeling to determine the potential of three types of MRI biomarkers (subcortical region volumes, cortical thickness, and resting-state functional connectivity) to identify individuals who were within 5 years of clinical motor diagnosis. An approach combining brain function and structure into a “polymarker” was reported to best distinguish between individuals who reached clinical motor diagnosis and those who did not. However, subcortical volumes alone had high accuracy for this classification (88%). The caudate, in particular, was highlighted by the authors as a strong predictor of motor diagnosis in clinical practice. In conclusion, there is considerable evidence indicating that vMRI can enhance predictive accuracy.

## MRI Brain Volume Measurements Have Robust Longitudinal Change Characteristics

**Figure 4:**
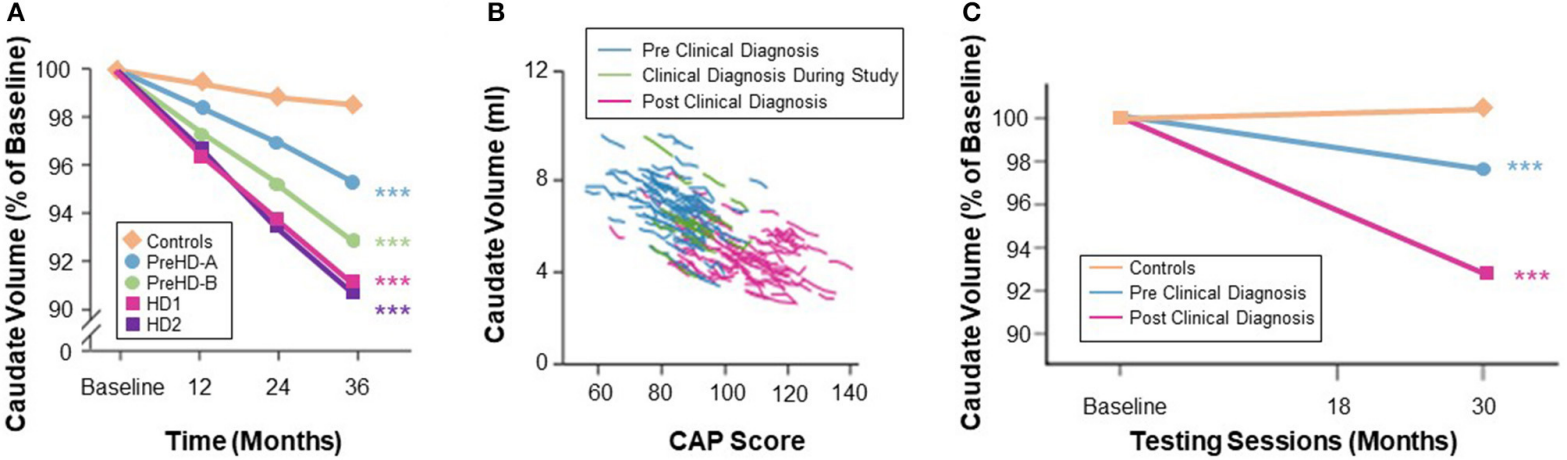
**(A)** Quantification of caudate volume loss in the TRACK-HD study showed near monotonic change from baseline to 12- and 24-month follow-up across the disease spectrum, with the rate of change increasing after clinical motor diagnosis (3). **(B)** Change in caudate volume with CAP score from TRACK-HD data (8). **(C)** Longer follow-up in the IMAGE-HD study also showed a greater degree of longitudinal change in the caudate volume in individuals with a clinical motor diagnosis (20).

Desirable characteristics for a biomarker of treatment response include an effect size for rate of change sufficient to detect a treatment-induced modification with a sample size smaller than that needed for standard clinical outcome measures. Observational studies have shown that vMRI measures have very good longitudinal performance characteristics in populations both before and after HD clinical motor diagnosis on a timescale typical for Phase 2 or 3 trials (1–2 years). The rate of longitudinal volume loss in the caudate and putamen has consistently been found to be higher in individuals with expanded CAG repeats than in matched controls, and to be more pronounced when both CAP score increases and functional capacity decreases (3, 18–20). Consistent with this, IMAGE-HD showed that longitudinal volume change in the caudate was the only measure among a range of multi-modal imaging features (including diffusion MRI) that discriminated between groups across different disease stages (i.e., far from clinical motor diagnosis [>15 years], close to diagnosis [ <15 years], and after diagnosis) (19). Estimates of annualized rates of volume loss in clinically-diagnosed participants (TFC ≥ 7) vary between 2 and 4% for the caudate and between 1 and 3% for the putamen (25, 60). Moreover, longitudinal data from PREDICT-HD showed that rates of volume loss in participants' putamen prior to clinical motor diagnosis were slightly higher than the rates of volume loss in the caudate, although both structures showed significantly elevated rates of change compared with controls, even in those furthest from expected clinical motor diagnosis (61). TRACK-HD (2) showed that at the most advanced disease stage studied (7 ≤ TFC ≤ 10) the caudate was approaching a constant rate of decline, and that annualized rates of change were consistent and stable over the 12-, 24-, and 36-month follow-up periods at both the group and individual levels (Figure 4).

**Figure 5:**
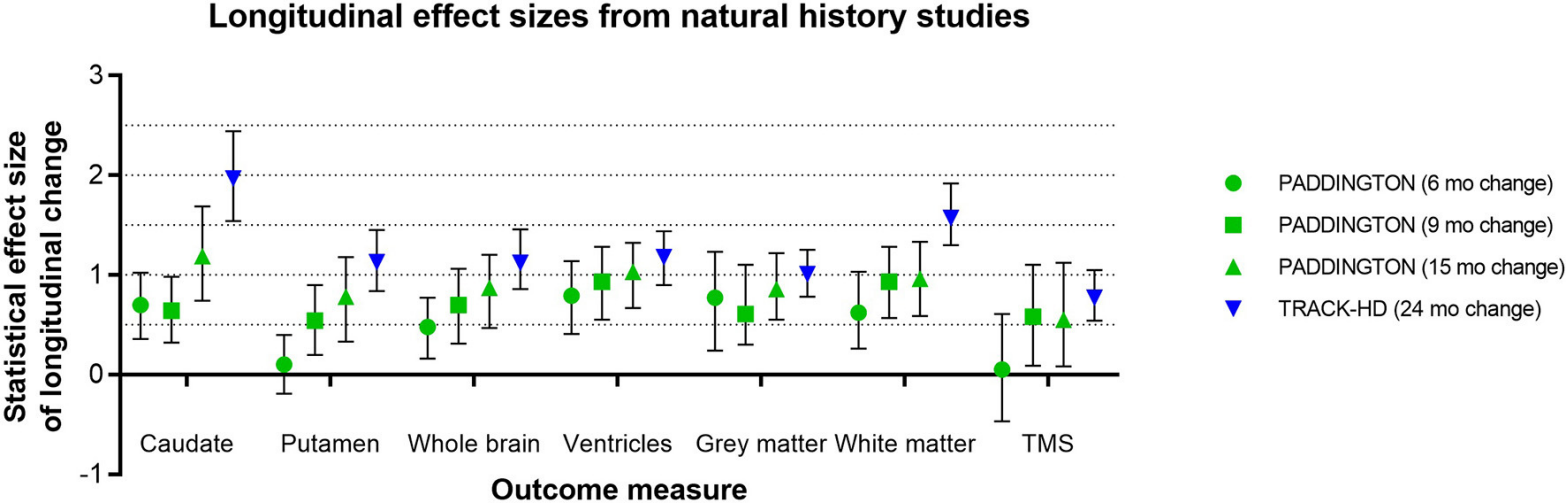
Representative longitudinal effect sizes (average change relative to standard deviation of the change) and their 95% confidence intervals for different time intervals in two observational studies [PADDINGTON (5) and TRACK-HD (3)]. Note that in the PADDINGTON study, the 9-month interval was the change between imaging at 6 and 15 months. The clinical status of the PADDINGTON population depicted here was primarily HD1 [*N* = 61 in total at baseline, of which *N* = 56 HD1 (TFC 11-13), *N* = 4 HD2 (TFC 7-10), and *N* = 1 HD3 (TFC 3-6); overall mean (SD) TFC was 11.7 (1.5)], and the TRACK-HD population was HD1 (*N* = 45 with TFC 11-13, 24-month follow-up).

Effect sizes for longitudinal change in caudate and putamen volumes have been consistently reported as larger than those for clinical outcome measures (15). Although many factors may influence these results—such as genetic and environmental modulators, image segmentation algorithm, statistical model, follow-up duration, sample size, and inclusion criteria—the effect size of caudate volume loss has been consistently reported as larger than that of putamen volume loss (3). IMAGE-HD similarly shows statistically greater longitudinal change, relative to controls, over 30 months for the caudate than the putamen both in participants before clinical motor diagnosis (UHDRS-TMS ≤ 5) and after clinical motor diagnosis (UHDRS-TMS > 5) (20). The PADDINGTON study examined longitudinal changes relative to healthy controls in clinically-diagnosed individuals (TFC ≥ 11) over shorter time intervals (i.e., 6, 9, and 15 months) and also found vMRI effect sizes to be larger than for clinical instruments and larger for the caudate than the putamen at each time interval (5) (Figure 5). Despite the differential changes between caudate and putamen, within-individual rates of striatal volume loss (both caudate and putamen) appear to decline linearly across participants regardless of clinical motor diagnosis status, but are more pronounced in individuals with a higher CAG-repeat length (8).

TRACK-HD, PREDICT-HD, and IMAGE-HD also provide a wealth of evidence for change over time in regions outside the striatum in individuals both before (2, 3, 62) and after (2, 3, 20) clinical motor diagnosis, with effect sizes equal to or greater than those for clinical instruments. Whole-brain volume loss, total white- and gray-matter volume losses, and increased ventricular volume reflect generalized cortical atrophy and degradation of white matter that structurally connects distinct brain regions. Voxel-wise analyses have also identified increasing losses of gray matter volume in the occipital, parietal, frontal and insular cortices as the disease advances (2, 63). A recent dynamic modeling approach that used 6 years of TRACK-HD and Track-On HD data revealed a temporal sequence of regional changes around the time of clinical motor diagnosis that involves not only the caudate and putamen but also cortical regions, including sensorimotor areas (64).

These robust longitudinal-change characteristics translate into attractive sample size estimates to detect putative treatment effects when using vMRI metrics as response biomarkers (3, 5, 65). Table 3 shows the estimated sample sizes per arm required to detect the specified change in a vMRI metric derived from power calculations from TRACK-HD and PADDINGTON data. Although in some cases the confidence intervals overlap, the sample sizes are all smaller than those required for clinical outcome measures obtained in the same studies. A systematic analysis of statistical powering considerations using TRACK-HD data concluded that whole-brain and caudate vMRI measures were substantially better powered than standard clinical outcome measures used in clinical trials (TMS and TFC), needing approximately one sixth of the sample size to detect the same degree of slowing (65). Stratification and adjustment for different covariates—such as age and CAG-repeat length (and their interaction), expected time to clinical motor diagnosis and baseline value, inclusion of scans at intermediate timepoints, and trial length—were all considered.

**Table 3:** The estimated required sample sizes per arm to detect the specified change in different vMRI metrics from power calculations on TRACK-HD (65) and PADDINGTON (5) data.

**Length**	**Study**	**Reference**	**Detection**	**Shoulson-Fahn disease stage**	**Sample size [95% CI]**
1 year	TRACK-HD	Frost et al. (65)	20% slowing of rate of whole-brain atrophy	TFC 11-13 & TFC 7-10	636 [454, 1,001]
1 year	TRACK-HD	Frost et al. (65)	40% slowing of rate of whole-brain atrophy	TFC 11-13 & TFC 7-10	159 [114, 251]
2 years	TRACK-HD	Frost et al. (65)	20% slowing of rate of whole-brain atrophy	TFC 11-13 & TFC 7-10	289 [211, 435]
2 years	TRACK-HD	Frost et al. (65)	40% slowing of rate of whole-brain atrophy	TFC 11-13 & TFC 7-10	73 [53, 109]
3 years	TRACK-HD	Frost et al. (65)	20% slowing of rate of whole-brain atrophy	TFC 11-13 & TFC 7-10	225 [158, 355]
3 years	TRACK-HD	Frost et al. (65)	40% slowing of rate of whole-brain atrophy	TFC 11-13 & TFC 7-10	57 [40, 89]
1 year	TRACK-HD	Frost et al. (65)	20% slowing of rate of caudate atrophy	TFC 11-13 & TFC 7-10	484 [363, 777]
1 year	TRACK-HD	Frost et al. (65)	40% slowing of rate of caudate atrophy	TFC 11-13 & TFC 7-10	121 [91–195]
2 years	TRACK-HD	Frost et al. (65)	20% slowing of rate of caudate atrophy	TFC 11-13 & TFC 7-10	197 [145, 350]
2 years	TRACK-HD	Frost et al. (65)	40% slowing of rate of caudate atrophy	TFC 11-13 & TFC 7-10	50 [37, 90]
3 years	TRACK-HD	Frost et al. (65)	20% slowing of rate of caudate atrophy	TFC 11-13 & TFC 7-10	144 [98, 284]
3 years	TRACK-HD	Frost et al. (65)	40% slowing of rate of caudate atrophy	TFC 11-13 & TFC 7-10	36 [25, 71]
6-month	PADDINGTON	Hobbs et al. (5)	50% slowing of rate of ventricular expansion	TFC ≥ 11	134 [64, 495]
9-month	PADDINGTON	Hobbs et al. (5)	50% slowing of rate of ventricular expansion	TFC ≥ 11	98 [51, 275]
15-month	PADDINGTON	Hobbs et al. (5)	50% slowing of rate of ventricular expansion	TFC ≥ 11	80 [48, 186]
6-month	PADDINGTON	Hobbs et al. (5)	50% slowing of rate of caudate atrophy	TFC ≥ 11	173 [81, 652]
9-month	PADDINGTON	Hobbs et al. (5)	50% slowing of rate of caudate atrophy	TFC ≥ 11	207 [87, 801]
15-month	PADDINGTON	Hobbs et al. (5)	50% slowing of rate of caudate atrophy	TFC ≥ 11	59 [30, 153]

## Relationships Between Longitudinal Change in Both Regional Brain Volumes and Clinical Outcome Measures

A biomarker closely associated with HD progression would be expected to exhibit measurable longitudinal change that correlates with changes in clinical instruments reflecting key aspects of the disease. Given that many clinical and imaging measures are associated with HD progression, these analyses should statistically control for CAP score and potentially other confounds such as study site, sex, and education. Such associations should be demonstrable in observational studies and, in interventional trials, a treatment-induced change in the biomarker would ideally be statistically associated with a concomitant change in the clinical marker(s). Moreover, it is important to understand which regional vMRI markers are associated with which clinical instruments in order to interpret treatment-induced effects (or lack thereof) appropriately. In principle, the robust longitudinal measurement characteristics of vMRI are well-suited to demonstrating such associations; however, the relatively greater variability in clinical instruments is likely to be the limiting factor, especially early in the disease course when the overall rate of change is slow.

**Figure 6:**
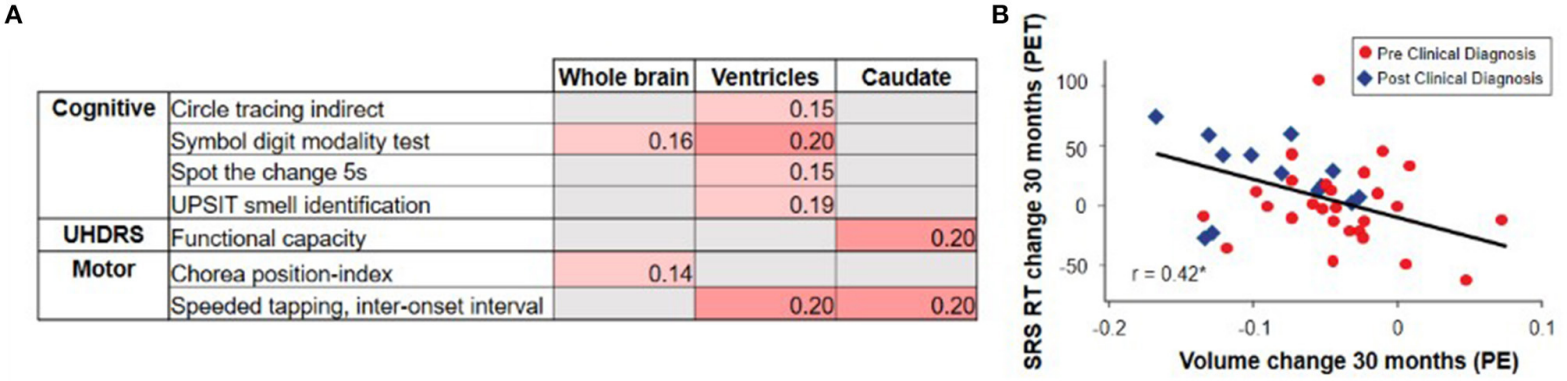
The TRACK-HD study showed **(A)** relatively weak and selective correlations between changes in vMRI metrics and changes in clinical outcomes over a 12-month interval (2). Partial correlations across participants with and without a clinical motor diagnosis, controlled for age, sex, education, and study site, nominally significant at *p* < 0.05 are shown. The IMAGE-HD study showed **(B)** that 30-month change in caudate volume is significantly correlated with 30-month change in reaction time (RT) during performance of a cognitive task that required shifting response set (SRS) (20) (i.e., greater volume loss was associated with longer reaction times [RTs]).

Overall, the evidence from observational studies relating change in clinical outcomes to change in vMRI metrics appears sparser than the cross-sectional relationships described earlier, with the strongest longitudinal relationships relating rates of brain volume loss to changes in cognitive and composite measures rather than purely motor measures (2, 19, 20, 64, 66) (Figure 6). For example, these studies have revealed relatively weak longitudinal associations between change in vMRI and change in UHDRS-TMS over a typical clinical trial time frame (1–2 years), despite the presence of a significant cross-sectional relationship between them. This finding may reflect that: longer follow-up time is needed for such a relationship to become apparent; the cross-sectional analyses capture larger temporal gaps between groups at different disease stages than the shorter time frames analyzed; group differences are easier to capture than individual progression curves; there is limited statistical power due to measurement variability and small sample sizes; and/or there are complex biological links between regional volume loss and outward presentation of HD clinical signs.

## Conclusions

Published reports and observational study datasets provide a promising evidentiary base for the utility of vMRI-based biomarkers in future HD interventional trials. However, the interpretability of vMRI-based biomarkers would be improved with a more complete understanding of how longitudinal change in vMRI metrics relates to change in clinical instruments on the timescale of typical clinical trials. An important next step will be to prospectively test the generalizability and robustness of the described longitudinal findings—particularly using more precise quantitative estimates of these relationships—across different datasets and image acquisition/analysis protocols. To be feasible, consensus for standardized practices for the use of vMRI in HD clinical research is necessary and will ultimately strengthen the interpretability of vMRI results. Recommendations to optimize the use of vMRI in HD clinical trials are discussed in another manuscript being prepared by this HD-RSC Biomarker Working Group, with a view to reaching consensus for best practice. Together, these actions will provide the HD research community with clear expectations for the use of vMRI in interventional trials.

## Author Contributions

KMK, AJS, APM and NG-K conceptualized the manuscript. KMK, AJS, ECT, DP, MFG, APM, RIS, and NG-K performed the literature search. KMK, AJS, ECT, DP, ECG, APM, RIS, and NG-K drafted the manuscript. All authors contributed to the article and approved the submitted version.

## Huntington's Disease Regulatory Science Consortium Coordinating Committee

Varun Aggarwal, Shazia Ali, Irina Antonijevic, Astri Arnesen, Nazem Atassi, Brian Beers, Beth Belluscio, Limor Ben Har, Angele Benard, Caroline Benn, Brian Bettencourt, Anu Bhattacharyya, Robi Blumenstein, Beth Borowsky, Bret Bostwick, Jackson Burton, Angelika Caputo, David Cooper, Brad Elmer, Rebecca Evans, Andrew Feigen, Terrence Fisher, Rebecca Fuller, Emily Gantman, Danielle Gartner, Michal Geva, Nellie Georgiou-Karistianis, Sandra Gonzalez, Adam Good, Mark Gordon, Jaya Goyal, Michael Hayden, Priyantha Herath, Steve Hersch, Jianying Hu, Elise Kayson, Eileen Koski, Bernhard Landwehrmeyer, Michelle Lax, Blair Leavitt, Dorothy Leong, Oren Levy, Enchi Liu, Jeff Long, Doug Macdonald, Jacqueline Major, Lahar Mehta, Tiago Mestre, Eric Miller, Christian Mueller, Catherine O'Riordan, Jennifer Panagoulias, Mike Panzara, Anne Pedata, Jennifer Petrillo-Billet, Dave Podskalny, Alisha Reader, Shelly Redman, Ralf Reilmann, Klaus Romero, Christopher Ross, Anne Rosser, Cristina Sampaio, Jan Samzelius, Scott Schobel, Adam Schwarz, Sudhir Sivakumaran, Jennie Socha, Glenn Stebbins, Julie Stout, Sarah Tabrizi, Emily Turner, Charles Venuto, Louise Vetter, Vissia Viglietta, Sarah Wahlstrom Helgren, Beth White, Ed Wild, George Yohrling, Maurice Zauderer.

## Conflict of Interest

KMK and RJ are full-time employees of IXICO. AJS is a full-time employee and shareholder of Takeda Pharmaceuticals, Ltd. DP and ECG are employed by CHDI Management to provide advisory services to CHDI Foundation. MFG is a full-time employee of Teva Pharmaceuticals. APM is a full-time employee of Wave Life Sciences, Ltd. RIS provides consultancy for IXICO. The remaining authors declare that the research was conducted in the absence of any other commercial or financial relationships that could be construed as a potential conflict of interest.

## Publisher's Note

All claims expressed in this article are solely those of the authors and do not necessarily represent those of their affiliated organizations, or those of the publisher, the editors and the reviewers. Any product that may be evaluated in this article, or claim that may be made by its manufacturer, is not guaranteed or endorsed by the publisher.

## Footnotes

^1^Consistent with the convention in the HD literature, in this article we will use the terms “striatum” or “striatal” as umbrella terms to refer to the dorsal striatal regions caudate and putamen.
