# Supplementary material for: Volumetric MRI-Based Biomarkers in Huntington's Disease: An Evidentiary Review
Source: Front Neurol. 2021 Sep 21;12:712555. doi: 10.3389/fneur.2021.712555 (PMC8490802; doi:10.3389/fneur.2021.712555)
Supplement: Supplementary file 1 [file Table_1.docx]

| **Study Name** | **Principal Investigator** | **Study Years** | **Disease Stage Relative to Clinical Diagnosis** | **Total Sample Size** | **MRI Sites** | **Field Strength** |
| --- | --- | --- | --- | --- | --- | --- |
| TRACK-HD | S. Tabrizi (UCL) | 2008 – 2014 | Before HD clinical motor diagnosis,  Clinically diagnosed HD  (with TFC 7-13) | 366 | 4 | 3T |
| TrackOn-HD | S. Tabrizi (UCL) | 2012 – 2014 | Before HD clinical motor diagnosis | 239 | 4 | 3T |
| IMAGE-HD | N. Georgiou-Karistianis (Monash) | 2008 – 2012 | Before HD clinical motor diagnosis,  Clinically diagnosed HD  (with TMS > 5) | 108 | 1 | 3T |
| PREDICT-HD | J. Paulsen (Iowa) | 2001 – 2012 | Before HD clinical motor diagnosis | 1314 | 33 | 3T |
| PADDINGTON | B. Landwehrmeyer (Ulm) | 2011 – 2013 | Clinically diagnosed HD  (with TFC ≥ 11) | 61 | 4 | 3T |

Supplementary Table 1: A selection of HD observational studies that include a longitudinal MRI component
