# Supplementary material for: Volumetric MRI-Based Biomarkers in Huntington's Disease: An Evidentiary Review
Source: Front Neurol. 2021 Sep 21;12:712555. doi: 10.3389/fneur.2021.712555 (PMC8490802; doi:10.3389/fneur.2021.712555)
Supplement: Supplementary file 2 [file Data_Sheet_1.docx]

# Supplementary information

## Observational studies

Several large-scale observational research studies have included longitudinal vMRI and are well-represented in the literature. As such, they have informed the design of subsequent clinical trials and provide a substantial substrate for potential future analysis and data mining. These studies are briefly summarized in the following (see also Table 1 in Supplementary Material).

**TRACK-HD** was a prospective observational biomarker study in participants before and shortly after HD clinical motor diagnosis. TRACK-HD assessed longitudinal 3T MRI, clinical, cognitive, quantitative motor, oculomotor and neuropsychiatric data collected at baseline, 12, 24, and 36 months. The study recruited 120 individuals with a CAG repeat expansion in the *HTT* gene (before HD clinical motor diagnosis), 123 early clinically diagnosed HD participants, and 123 controls. **Track-On HD** followed on from TRACK-HD, focusing on compensatory mechanisms in HD before clinical diagnosis. Baseline, 12-month and 24-month 3T vMRI, task-based and resting-state-functional MRI (fMRI), diffusion tensor imaging (DTI), clinical, cognitive, quantitative motor and neuropsychiatric data were collected from individuals with expanded CAG repeats and healthy controls. A total of 239 participants were recruited: 106 with expanded *HTT* CAG repeats (before HD clinical motor diagnosis), 22 clinically diagnosed HD participants, and 111 controls. Most participants came from Track-HD. The four sites in both studies were Leiden (Netherlands), London (UK), Paris (France), and Vancouver (Canada).

**IMAGE-HD** was a prospective multi-modal neuroimaging study with data collected at baseline, 18 and 30 months at the Melbourne site in Australia. A total of 108 participants were recruited, comprising of 36 healthy controls, 36 participants before HD clinical motor diagnosis (pre-HD) and 36 early clinically diagnosed HD (symptomatic; symp-HD) participants. Healthy controls were matched for age, sex, and IQ to the pre-HD individuals. Pre-HD and symp-HD participants were clinically assessed using the UHDRS motor assessment during each visit. CAG-repeat length ranged from 39 to 50 (42.2 ± 1.9 for pre-HD; 43.3 ± 2.5 for symp-HD). At each testing visit the protocol included 3T imaging with the collection of vMRI, DTI, and task-based fMRI (working memory and set-shifting tasks). At the final testing session (30 months), resting-state fMRI data were also collected. In addition, clinical, cognitive, motor, and neuropsychiatric data were collected at each visit.

**PREDICT-HD** studies aim to determine the earliest signs of HD and the timing of their onset. The completed studies, discussed here, involved blood tests, 3T vMRI, cognitive tests, and questionnaires about mood, thoughts, and lifestyle. This multicenter study recruited across 33 sites globally. All participants underwent elective DNA analyses for the CAG expansion in *HTT* and did not have a clinical motor diagnosis of HD at baseline [PreHD: CAG ≥39 repeats; healthy control (HC): CAG < 39 repeats]. A total of 1,013 PreHD and 301 HC were recruited. During the rolling enrollment in 2001 - 2012, 505 individuals were recruited across 24 sites (17 in the USA, 4 in Canada, 3 in Australia). The ongoing study (PREDICT-HD 3.0) is enrolling individuals both with and without *HTT* CAG expansion. Enrollment for participants with *HTT* gene expansion includes individuals before clinical motor diagnosis or individuals recently clinically diagnosed with HD.

**PADDINGTON** was a project aiming to develop and apply a range of biomarkers to support HD drug development. Work package 2 was an observational study with a goal to develop an assessment package to track the progression of clinically diagnosed HD in clinical trials. Sixty-one participants with early clinically diagnosed HD and 40 controls from four sites (London, UK; Paris, France; Leiden, Netherlands; Ulm, Germany) underwent vMRI at baseline, 6 months and 15 months, in addition to a medical interview, a neurological examination, cognitive assessments, and blood sampling (at a single timepoint).
